# Obstetric outcomes after IVF/ICSI treatment in women with endometriosis and/or adenomyosis diagnosed by ultrasonography: a prospective cohort study

**DOI:** 10.1093/humrep/deag084

**Published:** 2026-05-27

**Authors:** Sara Alson, Kristina Mattsson, Povilas Sladkevicius

**Affiliations:** Obstetric, Gynecological and Prenatal Ultrasound Research, Department of Clinical Sciences, Lund University, Malmö, Sweden; Department of Obstetrics and Gynecology, Skåne University Hospital, Malmö, Sweden; Reproductive Medicine Center, Skåne University Hospital, Malmö, Sweden; Department of Obstetrics and Gynecology, Skåne University Hospital, Malmö, Sweden; Perinatal and Cardiovascular Epidemiology, Department of Clinical Sciences, Lund University, Malmö, Sweden; Applied Epidemiology, Department of Clinical Sciences, Lund University, Lund, Sweden; Obstetric, Gynecological and Prenatal Ultrasound Research, Department of Clinical Sciences, Lund University, Malmö, Sweden; Department of Obstetrics and Gynecology, Skåne University Hospital, Malmö, Sweden

**Keywords:** assisted reproductive treatment, endometriosis, adenomyosis, ultrasound, obstetric and perinatal outcome

## Abstract

**STUDY QUESTION:**

Among women pregnant after IVF/ICSI, do obstetric outcomes differ for women diagnosed with endometriosis and/or adenomyosis according to definitions by International Deep Endometriosis (IDEA) and the revised Morphological Uterus Sonographic Assessment (MUSA) groups, compared to women without the disease?

**SUMMARY ANSWER:**

Women with endometriosis and/or adenomyosis have a higher risk of preterm birth, hypertensive disorders of pregnancy, placenta previa, antepartum hemorrhage, and pelvic pain, compared to women without.

**WHAT IS KNOWN ALREADY:**

Evidence regarding the impact of endometriosis and adenomyosis on obstetric outcomes remains inconsistent, largely due to heterogeneity in study design, diagnostic methods, disease criteria, and mode of conception. Therefore, clinical guidance on antenatal management for affected women remains limited.

**STUDY DESIGN, SIZE, DURATION:**

This was a prospective, observational cohort study of 1035 women who underwent up to three consecutive IVF/ICSI treatments at a university hospital. Published data suggest a preterm birth risk of 8–10% after IVF/ICSI without endometriosis, with higher rates in endometriosis and adenomyosis. Assuming 10% versus 20% preterm birth, 492 women were needed for 80% power. In total, 666 women gave birth to a child between January 2019 and April 2024. Of these, 607 (91.1%) women, for which the obstetric outcomes were known, were included in the study, while the remaining 59 (8.9%) were lost to follow-up.

**PARTICIPANTS/MATERIALS, SETTING, METHODS:**

Eligible for publicly funded IVF/ICSI treatments are non-smoking women aged 25 to ≤39 years, with a BMI of 18 to <30 kg/m^2^ and no previous children with the present partner. Women are entitled to up to three consecutive, publicly subsidized IVF/ICSI treatments, until the birth of the first child is achieved. All women underwent pretreatment ultrasound examination by an expert examiner, using the IDEA and revised MUSA definitions. Out of 607 included women, in total 144/607 (23.7%) women had endometriosis and/or adenomyosis. The primary outcome was preterm birth (delivery before completed 37 weeks gestation). Secondary outcomes included placenta previa, antepartum or postpartum hemorrhage, hypertensive disorders of pregnancy, gestational diabetes mellitus, caesarean section delivery, placental abruption, oligohydramnios, pelvic pain, and neonate small for gestational age, as well as outcomes stratified for women with different disease phenotypes. The adjusted relative risk for the different outcomes was calculated using modified Poisson regression analyses with robust error variances.

**MAIN RESULTS AND THE ROLE OF CHANCE:**

Preterm birth occurred in 27/144 (18.9%) women with endometriosis and/or adenomyosis compared to 53/463 (11.4%) in disease-free women, corresponding to an aRR 1.63 (95% CI, 1.06–2.49), *P* = 0.025. However, this was only significant for late preterm birth between gestational week 34 + 0–36 + 6 [21 (16%) vs 37 (8.0%), aRR 2.56 (95% CI, 1.26–5.21)] and not for earlier preterm birth. In addition, women with endometriosis and/or adenomyosis had an increased risk for placenta previa [13 (9.0%) vs 7 (1.5%), aRR 5.82 (95% CI, 2.32–14.6), *P* < 0.001], antepartum hemorrhage [17 (11.8%) vs 30 (6.5%), aRR 2.02 (95% CI, 1.19–3.41), *P* = 0.009], hypertensive disorders of pregnancy [19 (13.2%) vs 31 (6.7%), aRR 2.26 (95% CI, 1.29–3.97), *P* = 0.004] and pelvic pain [24 (16.7%) vs 40 (8.6), aRR 1.91 (95% CI, 1.21–3.01), *P* = 0.005] compared to disease-free women. There was a statistically non-significant tendency for an increased risk for developing oligohydramnios [10 (6.9%) vs 15 (3.3%), aRR 2.10, 95% CI, 0.98–4.48, *P* = 0.055] as well as delivering an SGA infant, [22 (15.3%) vs 45 (9.7%), aRR 1.58, 95% CI, 0.98–2.53, *P* = 0.059]. However, the risk for caesarean delivery, gestational diabetes mellitus, placental abruption, and postpartum hemorrhage was not increased.

**LIMITATIONS, REASONS FOR CAUTION:**

Excluding women aged ≥40 years or with a BMI ≥ 30 kg/m^2^ may limit the generalizability to other populations. The presence of superficial, peritoneal endometriosis was not accounted for. The study was powered for the primary outcome preterm birth in the total cohort and may have lacked sufficient power for the secondary outcomes. The composite endometriosis and/or adenomyosis group is dominated by isolated endometriosis, with relatively few women with combined disease. Women with adenomyosis only may represent a biologically and clinically distinct subgroup. Pooling them with endometriosis could potentially dilute or mask disease-specific associations.

**WIDER IMPLICATIONS OF THE FINDINGS:**

Our findings suggest that women with endometriosis and/or adenomyosis are at increased risk of common adverse pregnancy outcomes. Clinicians should recognize these risks when counseling affected women and consider tailored antenatal care.

**STUDY FUNDING/COMPETING INTEREST(S):**

This study was supported by regional research grants from Region Skåne, Sweden. There was no competing interest.

**TRIAL REGISTRATION NUMBER:**

Not applicable.

## Introduction

Endometriosis and adenomyosis are common, hormone-dependent gynecological disorders that affect ∼20–30% of women undergoing IVF or ICSI treatment. Evidence regarding the impact of these conditions on obstetric outcomes remains inconsistent, largely due to heterogeneity in study design, diagnostic methods, disease criteria, and mode of conception of the included pregnancies (Becker *et al.*, 2022). A key limitation of published data is the combination of women who conceived spontaneously with those who conceived through IVF/ICSI. This risks obscuring the findings, since IVF/ICSI pregnancies are also associated with an elevated risk of obstetric morbidity in themselves (Pandey *et al.*, 2012). While several studies and meta-analyses have reported increased risks of preterm birth (PTB), hypertensive disorders of pregnancy (HTD), placental abnormalities, and caesarean delivery among women with endometriosis or adenomyosis (Lalani *et al.*, 2018; Horton *et al.*, 2019; Busnelli *et al.*, 2024; Vendittelli *et al.*, 2025), others have not found such associations (Bean *et al.*, 2024), hence clinical guidance on antenatal management for women with endometriosis or adenomyosis remains limited. Therefore, prospective studies to better evaluate the potential obstetric risks for affected women are needed (Becker *et al.*, 2022).

Although endometriosis and adenomyosis have been studied separately, their combined impact on pregnancy outcomes is less well understood (Vercellini *et al.*, 2023). Given their shared pathophysiological features, with chronic inflammation, altered uterine contractility, and impaired myometrial and endometrial remodeling, it has been proposed that endometriosis and adenomyosis represent two distinct phenotypes within a single disease spectrum (Bulun *et al.*, 2023; Vercellini *et al.*, 2023; Donnez *et al.*, 2024; Cozzolino *et al.*, 2025). Accordingly, simultaneous evaluation of both conditions may provide a more comprehensive understanding of their contribution to adverse obstetric outcomes.

We have previously shown that most women with endometriosis or adenomyosis undergoing IVF/ICSI are unaware of their diagnosis, underscoring the need for systematic pre-treatment imaging for correct diagnosis and classification (Alson *et al.*, 2022, 2024c). Transvaginal ultrasonography (TVUS) is the first-line diagnostic tool for both disorders (Condous *et al.*, 2024), and the introduction of standardized imaging definitions by the International Deep Endometriosis Analysis (IDEA) (Guerriero *et al.*, 2016) and the Morphological Uterus Sonographic Assessment (MUSA) groups (Van den Bosch *et al.*, 2015, 2019). Harmsen *et al.* (2022) has greatly improved diagnostic consistency and comparability across studies.

Given the growing number of women with endometriosis and/or adenomyosis (E/A) conceiving through IVF/ICSI, a clearer understanding of the influence of E/A on obstetric outcomes is essential for individualized counseling and pregnancy management. The aim of this study was to evaluate obstetric outcomes after IVF/ICSI treatment in women with E/A diagnosed by standardized TVUS using the IDEA and revised MUSA definitions, respectively, prior to IVF/ICSI treatment.

## Materials and methods

### Study population

This was a prospective, observational cohort study of women who gave birth after undergoing up to three consecutive IVF/ICSI treatments between January 2019 and April 2024 at the Reproductive Medicine Center (RMC) at Skane University Hospital, Sweden. All eligible women who were referred to RMC for their first ART between December 2018 and May 2022 were asked to participate in the study. Eligible for publicly funded ART at RMC are women aged 25–39 years, who are non-smoking, with a normal BMI (18–30 kg/m^2^) and with more than 1 year’s infertility. Women are eligible for up to three consecutive IVF/ICSI treatments, until the birth of a living child is achieved. Only women who conceived and gave birth to a child were included in the study. Exclusion criteria were: (i) women undergoing oocyte donation or (ii) intrauterine inseminations; (iii) currently on hormonal treatment, as this may alter the ultrasonographic appearance of adenomyosis and last (iv) women with previous surgical destruction of superficial endometriotic lesions but without remaining lesions visible on TVUS, as they might otherwise have been misclassified as not having endometriosis.

### Ultrasound examination

All women underwent a systematic TVUS by the first author prior to starting their first IVF/ICSI treatment, using a Voluson E10 Expert (GE Medical Systems, Zipf, Austria) ultrasound machine, as previously described (Alson *et al.*, 2022, 2024c). The uterus was assessed for the presence of adenomyosis, using the revised MUSA definitions (Harmsen *et al.*, 2022). Direct, or pathognomonic, features of adenomyosis are lines and buds, myometrial cysts or hyperechogenic islands. Indirect features of adenomyosis (globular shape, fan-shaped shadowing, myometrial asymmetry, translesional vascularity, and an irregular or interrupted junctional zone (JZ)) are not conclusive of adenomyosis in the absence of direct features and were therefore not considered diagnostic. The presence of fibroids or uterine anomalies (classified according to the European Society of Human Reproduction and Embryology) (Grimbizis *et al.*, 2013) was documented. The adnexa and pelvis were assessed for endometriotic lesions. Deep endometriotic (DE) lesions were described using the IDEA terminology (Guerriero *et al.*, 2016). Endometriomas were defined as unilocular cysts with ground glass echogenicity (Van Holsbeke *et al.*, 2010). The presence of superficial endometriosis was not assessed for.

### IVF/ICSI treatment

The IVF/ICSI treatments have been previously described in detail (Alson *et al*., 2024a, b, 2025). Women were treated with either the GnRH agonist or antagonist protocol, with or without ultralong downregulation for 3–6 months (Liu *et al.*, 2021), depending on clinical characteristics and personal preferences. Depending on semen quality, mature oocytes were either injected or inseminated with sperm. Embryo transfer (ET) was performed either at the cleavage (Days 2–3) or at the blastocyst stage (Day 5), and single embryo transfer was standard practice. Embryos from patients unable to undergo fresh ET or surplus good-quality embryos (GQE) were cryopreserved on Days 5–6 after oocyte pickup. Frozen ET (FET) was performed in either natural or hormone replacement cycles. Luteal phase support was provided for 2 weeks with progesterone vaginal suppositories (Lutinus, Ferring, Lausanne, Switzerland). All fresh and frozen embryos from each treatment cycle were used until the birth of a child was achieved. Women were offered up to two additional IVF/ICSI treatment cycles if the birth of a living child after ≥22 gestational weeks was not achieved after the first treatment. Obstetric outcomes were collected from the hospital-based medical journal program Obstetrix 2.18.0 (Cerner, Sverige AB).

### Primary outcome

The primary outcome was PTB, defined as delivery prior to 37 gestational weeks (gws). Early PTB was a birth between ≥22–33 + 6 gws (i.e. extreme PTB < 28 gws and very PTB 28 to <34 gws) and late preterm was a birth in 34 + 0 to 36 + 6 gws.

### Secondary outcomes

Secondary outcomes were HTD, placenta previa (PP), placental abruption (PA), antepartum hemorrhage (APH), gestational diabetes mellitus (GDM), oligohydramnios, cesarean section (CS) (emergency or elective), small for gestational age (SGA), postpartum hemorrhage (PPH), and pelvic pain during pregnancy.

HTDs were either pregnancy-induced hypertension (PIH), defined as blood pressure >140 mmHg and 90 mmHg, or preeclampsia (PE), defined as PIH with significant proteinuria. PP was diagnosed if the placenta was partially or completely covering the internal cervical orifice on ultrasound and GDM in case of a positive oral glucose tolerance test. APH was significant antenatal bleeding, requiring observation in hospital, whereas PPH was defined as blood loss >1000 ml. PA was placental separation before delivery and oligohydramnios an amniotic fluid index < 50 mm, or single deepest pocket < 20 mm. SGA was weight below the 10th percentile for the gestational age on Swedish growth charts. Pelvic pain was examination in hospital due to abdominal or pelvic pain during pregnancy.

### Statistical analyses

To determine the number of participants required to detect a clinically relevant difference in PTB (<37 + 0 weeks) between women with and without E/A following IVF/ICSI, a sample size calculation was performed.

Published data indicate that the risk of PTB after ART is ∼8–10% in women without endometriosis (Stern *et al.*, 2020), and that women with endometriosis have a 30–50% relative increased risk of PTB (Breintoft *et al.*, 2022), which translate into absolute PTB rates of ∼12–15% among women with endometriosis. Women with adenomyosis have been shown to carry an even higher risk, with PTB rates reported at 18–25% (Horton *et al.*, 2019).

We assumed a 10% absolute difference in PTB between exposed and unexposed women (10% vs 20%). Using a two-sided alpha level of 0.05 and 80% power, we would need in total 492 pregnant women.

Data are presented as means with ±SD and medians (range) as appropriate. To compare percentages between groups, the chi-square or Fisher’s exact test was used. The Student’s *t*-test was used for comparing normally distributed data and the Mann–Whitney *U*-test to compare median values between groups. A *P*-value < 0.05 was considered statistically significant.

To estimate crude and adjusted relative risks (RR) for adverse obstetric outcomes in women with E/A, modified Poisson regression analyses with robust error variances were performed. RR are presented with 95% CIs. To identify potential confounders, a Directed Acyclic Graph was constructed based on *a priori* knowledge of contributing factors. A variable was included as a confounder if it met the set criteria of being associated with both the exposure (E/A) and the outcome. Included variables were age, BMI, uterine malformations, myoma, fertilization method (IVF vs ICSI) embryo stage at transfer, and type of embryo transfer (fresh or frozen).

Statistical analyses were performed using IBM SPSS Statistics for Windows, Version 29.0 (IBM Corp., released 2020, Armonk, NY, USA).

### Ethics

The study was conducted according to the Declaration of Helsinki for Medical Research. The study was approved by the Regional Ethical Review Board of Lund University, Lund, Sweden, on 11 September 2018, with a reference number 2018/555. Informed, written consent was obtained from all participants.

## Results

**Figure 1.**
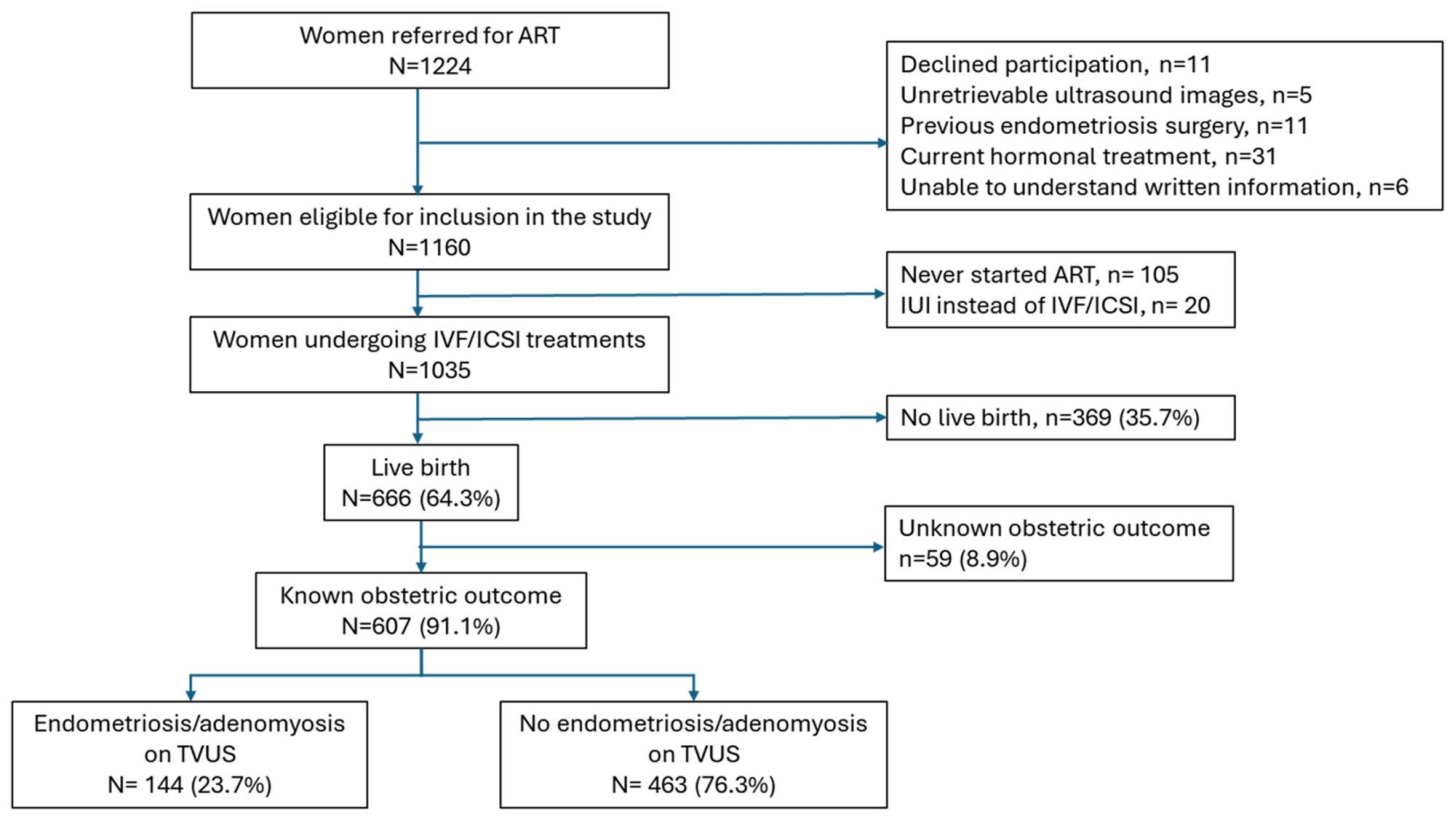
**Flowchart demonstrating the inclusion of participants in the study.** TVUS, transvaginal ultrasonography.

Among 1035 women who underwent 1–3 consecutive IVF/ICSI cycles, 666/1035 (64.3%) women gave birth to a child. Of these, 59/666 (8.9%) women were lost to follow-up, yielding a final sample of 607 women (Fig. 1).

In total, 144/607 (23.7%) women in the final study cohort had E/A. Of these, 123/607 (20.3%) women had endometriosis, whereas direct features of adenomyosis were present in 39/607 (6.4%) women. The composite E/A category was dominated by isolated endometriosis, with relatively few cases of combined disease and with concurrent endometriosis and adenomyosis present in merely 18/607 (3.0%) women. The sonographic phenotypes of endometriosis and adenomyosis are presented in Supplementary Table S1.

Of women with endometriosis on ultrasound examination, only 27/123 (22.0%) women had previously known disease, and none of them had previously had endometriosis surgery.

There was no difference in age or BMI between the two groups, or in the proportion of women with myoma or uterine malformations (Table 1). Adhesions in the pelvis were more common in women with E/A than in women without, Table 1.

**Table 1.** Background characteristics of the women included in the study, and for women with versus without endometriosis and/or adenomyosis on TVUS.

Parameter	Total cohort, n = 607	No endometriosis and/or adenomyosis n = 463	Endometriosis and/or adenomyosis, n = 144
Age, years	31.5 ± 3.7	31.4 ± 3.7	31.9 ± 3.7
BMI, kg/m^2^	23.9 ± 3.4	23.9 ± 3.3	23.7 ± 3.5
Length of subfertility, years	2.0 ± 1	2.0 ± 1	2.5 ± 1.9
s-AMH (pmol/l)	21 (19)	21 (19)	19 (17)
Mode of conception^a^			
IVF	233 (38.4)	175 (37.8)	58 (40.3)
ICSI	374 (61.6)	288 (62.2)	86 (59.7)
FET	184 (30.3)	140 (30.2)	44 (30.6)
Ultralong downregulation	18 (3.0)	2 (0.4)	16 (11.1)
Protocol			
Agonist	107 (17.6)	63 (13.6)	44 (30.6)
Antagonist	500 (82.4)	400 (86.4)	100 (69.4)
Previous termination of pregnancy	63 (10.4)	46 (9.9)	17 (11.8)
Previous miscarriage	68 (11.2)	54 (11.7)	14 (9.7)
Myoma	91 (15.0)	68 (14.7)	23 (16.0)
Myoma >5 cm	10 (1.6)	9 (1.9)	1 (0.7)
Uterine anomaly (unicornuate or bicorporeal)	13 (2.1)	10 (2.2)	3 (2.1)
Adhesions	284 (46.8)	148 (32.0)	136 (94.4)

s-AMH, serum antiMüllerian hormone; FET, frozen embryo transfer; TVUS, transvaginal ultrasonography.

a Of women undergoing either IVF or ICSI, some women underwent FET.

Values are given as n (%) of each group of women, mean ± SD or median (interquartile range).

PTB was more frequent among women with E/A (27/144, 18.9%) compared to women without (53/463, 11.4%), *P* = 0.043. However, the risk was increased only for late PTB, aRR 2.56 (95% CI, 1.26–5.21), *P* = 0.009, but not for early PTB (aRR 1.23, 95% CI, 0.49–3.11), Table 2. Extreme PTB < 33 gws occurred for only 10 (1.6%) women, 9 (1.9%) women without E/A, and 1 (0.7%) women with E/A.

**Table 2.** Adverse obstetric outcomes after IVF/ICSI treatment for women with and without endometriosis and/or adenomyosis.

Parameter	Total cohort, n = 607	No E/A, n = 463	E/A, n = 144	RR	95% CI	*P*-value	aRR^a^	95% CI	*P*-value
Preterm birth	80 (13.2)	53 (11.4)	27 (18.9)	1.64	1.07–2.5	0.023*	1.63	1.06–2.49	0.025*
Early^b^	22 (3.6)	16 (3.5)	6 (4.2)	1.21	0.48–3.05	0.679	1.23	0.49–3.11	0.656
Late^c^	58 (9.6)	37 (8.0)	21 (14.6)	2.61	1.28–5.3	0.008*	2.56	1.26–5.21	0.009*
Caesarean section	135 (22.2)	99 (21.4)	36 (25.0)	1.17	0.84–1.63	0.357	1.14	0.82–1.59	0.451
Placenta previa	20 (3.3)	7 (1.5)	13 (9.0)	5.97	2.43–14.7	<0.001*	5.82	2.32–14.6	<0.001*
Small for gestational age	67 (11.0)	45 (9.7)	22 (15.3)	1.57	0.98–2.53	0.062	1.58	0.98–2.53	0.059
Gestational diabetes mellitus	26 (4.3)	20 (4.3)	6 (4.2)	0.97	0.40–2.36	0.937	0.94	0.38–2.33	0.901
Hypertensive disorders of pregnancy^d^	50 (8.2)	31 (6.7)	19 (13.2)	1.97	1.15–3.38	0.014*	1.98	1.15–3.41	0.014*
Pregnancy induced hypertension	19 (3.1)	11 (2.4)	8 (5.6)	2.34	0.96–5.70	0.062	2.42	1.01–5.83	0.048*
Preeclampsia	31 (5.1)	20 (4.3)	11 (7.6)	1.77	0.87–3.60	0.116	1.75	0.84–3.62	0.133
Placental abruption	11 (1.8)	8 (1.7)	3 (2.1)	1.20	0.32–4.48	0.783	1.16	0.31–4.37	0.828
Oligohydramnios	25 (4.1)	15 (3.3)	10 (6.9)	2.14	0.99–4.67	0.055	2.10	0.98–4.48	0.055
Postpartum hemorrhage	61 (10.4)	43 (9.3)	18 (12.5)	1.35	0.80–2.26	0.260	1.35	0.80–2.27	0.256
Antepartum hemorrhage	47 (7.7)	30 (6.5)	17 (11.8)	1.82	1.03–3.20	0.038*	2.02	1.19–3.41	0.009*
Pelvic pain	64 (10.5)	40 (8.6)	24 (16.7)	1.90	1.12–3.04	0.007*	1.91	1.21–3.01	0.005*

aRR, adjusted relative risk; E/A, endometriosis and/or adenomyosis.

a Adjusted for age and BMI.

b Delivery between ≥22–33 + 6 gestational weeks.

c Delivery between 34 + 0 and 36 + 6 gestational weeks.

d Pregnancy-induced hypertension or preeclampsia. Numbers are given as n (%). Some women may have had more than one adverse obstetric outcome. RR were calculated using a modified Poisson regression analysis.

* *P* < 0.05 is statistically significant.

Women with E/A had an almost 6 times higher risk of PP compared to women without, aRR 5.82 (95% CI, 2.32–14.6), *P* < 0.001. They also had a higher risk of APH, aRR 2.02 (95% CI, 1.19–3.41), *P* = 0.009, Table 2.

The risk of HTD was increased, aRR 1.98 (95% CI, 1.15–3.41), *P* = 0.014, Table 2. There was a statistically non-significant tendency for an increased risk for developing oligohydramnios (aRR 2.10, 95% CI, 0.98–4.48, *P* = 0.055) as well as delivering an SGA infant (aRR 1.58, 95% CI, 0.98–2.53), *P* = 0.059. When stratifying the results for women with only endometriosis without adenomyosis (n = 105, 17.3%), a higher proportion of women had an SGA infant compared to women without (18.1% vs 9.7%, *P* = 0.014), Table 3. Women with E/A had an increased risk of pelvic pain, aRR 1.91 (95% CI, 1.21–3.01), *P* = 0.005, Table 2.

**Table 3.** Adverse obstetric outcomes after IVF/ICSI treatment for women with endometriosis, without adenomyosis.

Parameter	No endometriosis n = 463	Endometriosis n = 105	*P*-value
Preterm birth	53 (11.4)	19 (18.1)	0.065
Early^a^	16 (3.5)	2 (1.9)	0.55
Late^b^	37 (8.0)	17 (2.1)	0.010*
Caesarean section	99 (21.4)	22 (21.0)	0.923
Placenta previa	7 (1.5)	9 (8.6)	<0.001*
Small for gestational age	45 (9.7)	19 (18.1)	0.014*
Gestational diabetes mellitus	20 (4.3)	3 (2.9)	0.783
Hypertensive disorders of pregnancy	31 (6.7)	13 (12.4)	0.049*
Pregnancy induced hypertension	11 (2.4)	4 (3.8)	0.41
Preeclampsia	20 (4.3)	9 (8.6)	0.076
Placental abruption	8 (1.7)	3 (2.9)	0.436
Oligohydramnios	15 (3.2)	7 (6.7)	0.155
Postpartum hemorrhage	43 (9.3)	16 (15.2)	0.071
Antepartum hemorrhage	30 (6.5)	14 (13.3)	0.018*
Pelvic pain	40 (8.6)	21 (20.0)	<0.001*

HT, hypertension; HTD, hypertensive disorder (HT or PE); SGA, small for gestational age.

a Delivery between ≥22–33 + 6 gestational weeks.

b Delivery between 34 + 0 and 36 + 6 gestational weeks. Numbers are given as n (%). Some women may have had more than one adverse obstetric outcome. Comparison between groups was made with the chi-square test or Fischer’s exact test.

* *P* < 0.05 is considered statistically significant.

There were no significant associations for CS, GDM, placental abruption, or PPH, Table 2.

The adverse obstetric outcomes for women with only endometriosis (n = 105, 17.3%) or only adenomyosis (n = 21, 3.5%) are presented in Tables 3 and 4. Due to small groups, results should be interpreted with caution.

**Table 4.** Adverse obstetric outcomes after IVF/ICSI treatment for women with adenomyosis, without endometriosis.

Parameter	No adenomyosis n = 463	Adenomyosis n = 21	*P*-value
Preterm birth	53 (11.4)	4 (19.0)	0.294
Early^a^	16 (3.5)	1 (4.8)	0.536
Late^b^	37 (8.0)	3 (14.3)	0.297
Caesarean section	99 (21.4)	9 (42.9)	0.030*
Placenta previa	7 (1.5)	3 (14.3)	0.007*
Small for gestational age	45 (9.7)	1 (4.8)	0.709
Gestational diabetes mellitus	20 (4.3)	2 (9.5)	0.246
Hypertensive disorders of pregnancy	31 (6.7)	2 (9.5)	0.647
Pregnancy induced hypertension	11 (2.4)	1 (4.8)	0.416
Preeclampsia	20 (4.3)	1 (4.8)	0.614
Placental abruption	8 (1.7)	0 (0)	1.0
Oligohydramnios	15 (3.2)	2 (9.5)	0.165
Postpartum hemorrhage	43 (9.3)	1 (4.8)	0.710
Antepartum hemorrhage	32 (6.9)	3 (14.3)	0.642
Pelvic pain	40 (8.6)	1 (4.8)	1.0

a Delivery between ≥22–33 + 6 gestational weeks.

b Delivery between 34 + 0 and 36 + 6 gestational weeks.

Numbers are given as n (%). Some women may have had more than one adverse obstetric outcome. Comparison between groups was made with the chi-square test or Fischer’s exact test.

* *P* < 0.05 is considered statistically significant.

The subgroup analysis of women with different phenotypes of endometriosis or adenomyosis was limited due to very few women in some groups, why results are presented as Supplementary Material (Supplementary Table S2). Subgroup analyses of women with E/A showed no significant differences in PTB or secondary outcomes stratified by ultralong GnRH downregulation. PTB was 3/16 (18.8%) in women with ultralong treatment, versus 1/12 (8.3%) in women without; *P* = 0.613 (Supplementary Table S3). However, these results should be interpreted with caution due to the small sample size.

## Discussion

In this prospective cohort study of women conceiving through IVF/ICSI, those diagnosed with endometriosis and/or adenomyosis had a higher risk of several adverse obstetric outcomes, including late PTB, hypertensive disorders of pregnancy, placenta previa, antenatal hemorrhage, and pelvic pain, compared to women without these conditions.

To the best of our knowledge, this is the first prospective study to evaluate obstetric outcomes in a cohort of IVF/ICSI pregnancies where all women underwent standardized pre-treatment ultrasound screening for endometriosis and adenomyosis, using the IDEA and revised MUSA definitions, respectively (Guerriero *et al.*, 2016; Harmsen *et al.*, 2022).

Comparison with previous studies is hampered by different study designs, study populations, and diagnostic methods. Earlier studies have often relied on surgical diagnoses, non-standardized ultrasound assessments, or self-reported history, introducing misclassification bias and contributing to inconsistent results. Many studies were retrospective and used diagnoses from databases or national registers, during time periods when high-resolution ultrasound machines and the same and clear diagnostic criteria that are used today were lacking (Ibiebele *et al.*, 2022; Vendittelli *et al.*, 2025). One prospective study that evaluated the obstetric outcomes for women with endometriosis, and in which all women were diagnosed by systematic ultrasound using the IDEA definitions, found similar odds of PTB, PP, or APH for women with or without endometriosis (Bean *et al.*, 2024). However, they included both women who conceived naturally and after ART. Since IVF/ICSI pregnancies themselves are associated with an elevated baseline risk of adverse obstetric outcomes (Pandey *et al.*, 2012), and women with endometriosis who conceive through ART could represent a subgroup at particularly high risk for pregnancy complications (Helmerhorst *et al.*, 2004), failure to stratify by conception mode may obscure disease-specific effects. Moreover, only women who sought care due to early pregnancy complications were included in the study by Bean *et al.*, which may have introduced bias.

### PTB and abnormal placentation

The increased risk of PTB among women with endometriosis and/or adenomyosis is consistent with earlier studies (Exacoustos *et al.*, 2016; Farland *et al.*, 2019; Ibiebele *et al.*, 2022; Kato *et al.*, 2024; Liu *et al.*, 2025). Chronic inflammation, abnormal uterine contractility, and impaired cervical competence are proposed mechanisms linking endometriosis and/or adenomyosis to premature delivery (Goldenberg *et al.*, 2008; Petraglia *et al.*, 2012). In our cohort, the excess risk was confined to late PTB, which contrasts previous data by Glavind *et al.*, who reported an increased OR for PTB, with the highest risk for very PTB (aOR 1.91, 95% CI, 1.16–3.15) irrespective of mode of conception (Glavind, Forman *et al.*, 2017). Similar data were reported in a recent retrospective cohort study of 368 935 women, in which women with endometriosis and/or adenomyosis had a higher aRR for preterm delivery <37 weeks (1.40, 95% CI, 1.18–1.67) and <33 weeks (1.53, 95% CI, 1.08–2.16) (Vendittelli *et al.*, 2025). However, PTB < 33 gws was a very rare event in our cohort, occurring for only 10 (1.6%) women. Our study may have lacked sufficient power to detect differences in this subgroup, and a larger cohort may be needed to investigate this association.

The increased risk of PTB in IVF/ICSI conceived pregnancies may in some cases be iatrogenic (Cavoretto *et al.*, 2022). Possibly, the increased risk of late PTB observed for women with endometriosis and/or adenomyosis in our cohort may be secondary to obstetric interventions prompted by placental or bleeding complications, as well as HTD. The associations between endometriosis and/or adenomyosis with PP and APH that we observed align with findings from previous studies and large meta-analyses (Stephansson *et al.*, 2009; Harada *et al.*, 2016; Berlac *et al.*, 2017; Saraswat *et al.*, 2017; Lalani *et al.*, 2018; Horton *et al.*, 2019; Busnelli *et al.*, 2024; Kato *et al.*, 2024; Liu *et al.*, 2025). Aberrant uterine peristalsis and a disrupted junctional zone in endometriosis and/or adenomyosis may displace blastocyst implantation sites toward the lower uterine segment (Kunz *et al.*, 2000). Additionally, pregnancies conceived through ART already carry an inherent risk of abnormal placentation (Qin *et al.*, 2016) which may amplify the effect.

### Hypertensive disorders of pregnancy

Women with endometriosis and/or adenomyosis had an increased risk for developing HTD. This is consistent with current theories of an association between poor placentation with defective spiral artery remodeling, endothelial inflammation, and increased maternal vascular resistance and blood pressure in endometriosis and/or adenomyosis (Brosens *et al.*, 2002; Harada *et al.*, 2022). Several previous systematic reviews and meta-analyses describe a small increase in the risk of PIH in women with endometriosis and/or adenomyosis (Lalani *et al.*, 2018; Perez-Lopez *et al.*, 2018; Breintoft *et al.*, 2021; Wang *et al.*, 2024) and a small or non-significant increase in the risk of PE in women with endometriosis (Lalani *et al.*, 2018; Perez-Lopez *et al.*, 2018; Qu *et al.*, 2022). Others did not find any significant difference in the incidence of PE (Perez-Lopez *et al.*, 2018) or HTD/PE (Leone Roberti Maggiore *et al.*, 2016) between women with or without endometriosis.

### SGA infants

There was a tendency toward an increased risk for delivering an SGA infant for women with endometriosis and/or adenomyosis, even if this did not reach statistical significance. While abnormal placentation and uterine contractility may contribute to fetal growth restriction, it is possible that the use of ultralong GnRH protocols may reduce inflammation and improve placental function. Previous evidence regarding fetal growth restriction in these populations remains inconsistent, with some meta-analyses linking endometriosis or adenomyosis to higher SGA risk (Leone Roberti Maggiore *et al.*, 2016; Nirgianakis *et al.*, 2021) and others showing no difference (Lalani *et al.*, 2018; Horton *et al.*, 2019). Our results indicate that disease phenotype, localization, and treatment strategy may influence fetal growth outcomes, but larger studies are needed to confirm this.

### Pelvic pain

Seeking medical care due to pelvic pain during pregnancy was more common in women with endometriosis and/or adenomyosis than women without. Possibly, this could be due to the presence of adhesions in the pelvis, which was more common in women with endometriosis and/or adenomyosis, or due to abnormal uterine contractions. We could also speculate that lower pelvic and perineal pain during pregnancy in some women could be associated with decidualized deep endometriosis nodules, an observation that should be confirmed in larger studies.

### Gestational diabetes mellitus and postpartum hemorrhage

No association was observed between endometriosis and/or adenomyosis and GDM, which corroborates with previous meta-analyses (Lalani *et al.*, 2018; Farland *et al.*, 2019). Because GDM risk increases with age and obesity (Chu *et al.*, 2007), the exclusion of older and obese women in our study likely contributed to our results. Moreover, previous data are contradictory, and in ART populations, the link between endometriosis and GDM appears attenuated (Lalani *et al.*, 2018; Farland *et al.*, 2019).

Similarly, we found no increased risk of PPH, in contrast to some meta-analyses (Lalani *et al.*, 2018; Horton *et al.*, 2019; Breintoft *et al.*, 2021) but in agreement with several large cohort studies (Saraswat *et al.*, 2017; Yi *et al.*, 2020; Velez *et al.*, 2022). PPH is usually defined as cumulative blood loss of greater than or equal to 1000 ml, whereas some studies consider it as bleeding >500 ml, which together with subjective estimation methods of blood volume, likely account for inconsistencies across studies.

### Strengths and limitations

The main strength of this study is the prospective design, with systematic pre-treatment ultrasound examinations by an expert examiner, using current international definitions. This ensures consistent and reproducible phenotyping of disease as well as accurate detection of both symptomatic and asymptomatic disease. As the presence of endometriosis was previously unknown in 75% of women with endometriosis in our study (Alson *et al.*, 2022), we minimized the risk of misclassification, which is otherwise an inherent risk. Moreover, we were also able to diagnose adenomyosis. However, this approach may limit generalizability to clinics without similar expertise or equipment. Another important strength is performing the study among women who all underwent IVF/ICSI treatment, lowering the risk of mediation bias due to an increased prevalence of ART in women with endometriosis and/or adenomyosis.

Nevertheless, several limitations must be acknowledged. Laparoscopic and histopathological confirmation were not available, which may have led to underdiagnosis of superficial disease. However, systematic TVUS using the IDEA approach has high concordance with laparoscopy (Goncalves *et al.*, 2021; Leonardi *et al.*, 2022). Surgical confirmation would neither have been feasible nor ethically justified, as laparoscopy is not part of routine infertility work-up (Becker *et al.*, 2022) and hysterectomy is not appropriate for subfertile women wishing to conceive, which is a limitation shared by most similar studies. Some misclassifications of adenomyosis are still possible, as we adhered strictly to the revised MUSA criteria, defining adenomyosis as the presence of direct features to ensure high specificity while capturing early-stage or mild cases (Harmsen *et al.*, 2022). However, in some cases, it is possible that indirect features may indeed indicate disease. Conversely, a small number of women may have been incorrectly categorized as affected, an inherent limitation of current MUSA criteria, which should be considered in a future revision of these definitions.

Combining women with endometriosis and adenomyosis into a single group may be considered a limitation, particularly as the composite category is dominated by isolated endometriosis with relatively few cases of concurrent disease. The rationale for combining endometriosis and adenomyosis stems from their high co-occurrence and proposed shared pathophysiology. In our cohort, a large proportion of women with endometriosis exhibited concurrent JZ alterations (Alson *et al.*, 2024c). Although not always conclusive of adenomyosis according to revised MUSA definitions (Harmsen *et al.*, 2022), JZ alterations are frequently observed on Magnetic Resonance Imaging in women with pelvic endometriosis (Kunz *et al.*, 2005; Larsen *et al.*, 2011) and may represent early manifestations of adenomyosis (Maubon *et al.*, 2010). Some evidence suggests that adenomyosis develops earlier in women with endometriosis (Kunz *et al.*, 2007), where indirect features might precede direct signs as part of a premature uterine ageing process (Larsen *et al.*, 2011). Given this, a composite category was deemed appropriate for evaluating obstetric risks.

However, while endometriosis and adenomyosis are often comorbid, adenomyosis is increasingly recognized as a clinically distinct entity with specific uterine, placentation, and vascular implications, including JZ disruption and altered deep placentation, that may differ in magnitude, or even direction, from those associated with endometriosis alone. Consequently, women with isolated adenomyosis may represent a biologically unique subgroup, and pooling these with endometriosis could potentially dilute disease-specific associations or obscure distinct obstetric risk profiles. These potentially distinct pathophysiological pathways from endometriosis could independently influence pregnancy outcomes and should be considered when discussing obstetric risks. While our subgroup analyses suggested differences in outcomes, such as the risk of SGA in isolated endometriosis, the study was underpowered to draw definitive conclusions regarding these nuances. The inherent power constraints, particularly in the adenomyosis subgroup, must be acknowledged. Future, larger-scale studies are warranted to evaluate whether the obstetric risks associated with isolated endometriosis and isolated adenomyosis are truly divergent.

Our findings may primarily be extrapolated to patients with non-operated endometriosis. However, previous studies have shown that surgical excision does not necessarily eliminate the risk of obstetric complications (Nirgianakis *et al.*, 2018). Whether the risks observed in our study would be further compounded or mitigated by prior surgery remains an area for future investigations.

In our cohort, some women received ultralong GnRH agonist downregulation prior to treatment. Prolonged suppression of estrogen-dependent disease activity may theoretically reduce inflammation, angiogenesis, and abnormal uterine contractility and potentially improve the uterine environment during implantation and early placentation. It could therefore be hypothesized that such pretreatment might mitigate obstetric complications associated with these conditions. However, most studies evaluate the effect of GnRH downregulation on ART outcomes, while data specifically addressing obstetric outcomes are scarce (Becker *et al.*, 2022). In the present study, no differences were observed between women with or without ultralong pretreatment in subgroup analyses. However, these analyses were limited by small sample sizes and low event rates, therefore no reliable assessment could be made.

The study cohort was relatively uniform, as eligibility criteria for publicly funded IVF at RMC exclude women <25 years or ≥40 years and those with a BMI ≥ 30 kg/m^2^. This could limit generalizability to broader populations, particularly older or multiparous women, in whom adenomyosis is more prevalent. Finally, the sample size may not have been sufficient to detect differences in rare outcomes such as severe PE or very PTB.

## Conclusion

In conclusion, this study finds that women with endometriosis and/or adenomyosis pregnant after IVF/ICSI are at additional increased risk of adverse pregnancy outcomes compared to women without endometriosis and/or adenomyosis giving birth after IVF/ICSI treatment. Clinicians should recognize these risks when counseling affected women and consider tailored antenatal care, including early screening for hypertensive disorders and increased surveillance for placental complications. However, it should be acknowledged that the underlying pathophysiological mechanisms of adenomyosis may differ from those of endometriosis, which should be considered when obstetric risks are discussed. Larger studies are needed to evaluate possible differences in obstetric outcomes between women with either only endometriosis or only adenomyosis.

## Supplementary Material

deag084_Supplementary_Table_S1

deag084_Supplementary_Table_S2

deag084_Supplementary_Table_S3

## Data Availability

The data underlying this article cannot be shared publicly due to ethical reasons and for the privacy of the participants. The data will be shared at reasonable requests to the corresponding author.
