# Supplementary material for: Obstetric outcomes after IVF/ICSI treatment in women with endometriosis and/or adenomyosis diagnosed by ultrasonography: a prospective cohort study
Source: Hum Reprod. 2026 May 27;41(8):1367–76. doi: 10.1093/humrep/deag084 (PMC13429875; doi:10.1093/humrep/deag084)
Supplement: deag084_Supplementary_Table_S1 [file deag084_supplementary_table_s1.pdf]

Human Reproduction, 2026, 1–1
https://doi.org/10.1093/humrep/deag084
Supplementary Data

human reproduction
OXFORD

Supplementary Table S1. Disease phenotypes for women with endometriosis and/or adenomyosis.

Parameter	Endo and/or adeno, n = 144
Endometriosis	123 (85.4)
Endometrioma	58 (40.3)
DE	100 (69.4)
DE and endometrioma	35 (24.3)
DE in sacrouterine ligaments	63 (43.8)
DE in Bowel	39 (27.1)
DE in vaginal wall	18 (12.5)
DE in urinary bladder	0 (0)
DE in rectovaginal septum	22 (15.3)
Adhesions	136 (94.4)
Direct features of adenomyosis	39 (27.1)
≥2 features (direct)	14 (9.7)
≥2 features (direct or indirect)	58 (40.3)
Myometrial cysts	15 (10.4)
Lines and buds	30 (20.8)
Hyperechogenic islands	17 (11.8)
Direct and indirect features of adenomyosis	131 (91.0)
Only indirect features of adenomyosis	92 (63.9)
Type of adenomyosis[a]	
Focal	21 (14.6)
Diffuse	10 (6.9)
Mixed	8 (5.6)
Myometrial layer with adenomyosis[a]	
Inner	30 (20.8)
Middle	7 (4.9)
Outer	2 (1.4)
Endometriosis and direct features of adenomyosis	18 (12.5)
Endometriosis and indirect features of adenomyosis	50 (34.7)

DE = Deep endometriosis.
[a] Out of women with direct features of adenomyosis. Numbers are given as n (%). Some women may have had endometriosis in multiple locations, or several different types of features of adenomyosis. Endo, endometriosis; adeno, adenomyosis.
