# Supplementary material for: Obstetric outcomes after IVF/ICSI treatment in women with endometriosis and/or adenomyosis diagnosed by ultrasonography: a prospective cohort study
Source: Hum Reprod. 2026 May 27;41(8):1367–76. doi: 10.1093/humrep/deag084 (PMC13429875; doi:10.1093/humrep/deag084)
Supplement: deag084_Supplementary_Table_S2 [file deag084_supplementary_table_s2.pdf]

Human Reproduction, 2026, 1–1
https://doi.org/10.1093/humrep/deag084
Supplementary Data

human reproduction

OXFORD

Supplementary Table S2. Adverse obstetric outcomes for women with different phenotypes of endometriosis and adenomyosis.

Parameter	EOMA[a] n = 17	*P*-value	DE[b] n = 55	*P*-value	EOMA and DE[c] n = 33	*P*-value	Indirect[d] n = 60	*P*-value	Direct and indirect[e] n = 21	*P*-value
Preterm birth	2 (11.8)	1.0	10 (18.2)	0.149	7 (21.2)	0.17	9 (15.0)	0.354	4 (19.0)	0.294
Early[f]	0	NA	1 (1.8)	0.607	1 (3.0)	0.419	2 (3.3)	0.329	1 (4.8)	0.361
Late[g]	2 (11.8)	0.64	9 (16.4)	0.047*	6 (18.2)	0.115	7 (11.7)	0.303	3 (14.3)	0.404
Cesarean section	1 (5.9)	0.545	13 (23.6)	0.701	7 (21.2)	0.99	20 (33.3)	0.016*	9 (42.9)	0.030*
Placenta previa	1 (5.9)	0.252	6 (10.4)	0.001*	2 (6.1)	0.237	0	0.602	3 (14.3)	0.007*
Small for gestational age	1 (5.9)	1.0	10 (18.2)	0.054	8 (24.2)	0.023*	5 (8.3)	0.698	1 (4.8)	0.709
Gestational diabetes mellitus	0	1.0	1 (1.8)	0.714	2 (6.1)	0.637	2 (3.3)	1.0	2 (9.5)	0.246
Hypertensive disorders of pregnancy	2 (11.8)	0.274	7 (12.7)	0.051	4 (12.1)	0.279	7 (11.7)	0.068	2 (9.5)	0.416
Pregnancy induced hypertension	1 (5.9)	0.355	3 (5.5)	0.174	0	NA	2 (3.3)	0.642	1 (4.8)	0.363
Preeclampsia	1 (5.9)	0.539	4 (7.3)	0.307	4 (12.1)	0.059	5 (8.3)	0.601	1 (4.8)	0.614
Placental abruption	1 (5.9)	0.280	1 (1.8)	1.0	1 (3.0)	0.486	0	0.604	0	0.363
Oligohydramnios	1 (5.9)	0.444	5 (9.1)	0.033	1 (3.0)	1.0	1 (1.7)	0.705	2 (9.5)	0.165
Postpartum hemorrhage	2 (11.8)	0.668	7 (12.7)	0.466	7 (21.2)	0.068	7 (11.7)	0.496	1 (4.8)	0.710
Antepartum hemorrhage	3 (17.6)	0.104	8 (14.5)	0.049*	3 (9.1)	0.735	2 (3.3)	0.404	2 (9.5)	0.642
Pelvic pain	3 (17.6)	0.197	12 (21.8)	0.003*	6 (18.2)	0.158	4 (6.7)	0.633	1 (4.8)	1.0

EOMA = Endometrioma.

[a] Women with direct features or DE are excluded.
[b] Women with endometrioma or direct features are excluded.
[c] Women with direct features are excluded.
[d] Women with endometriosis or direct features are excluded.
[e] Women with both direct and indirect features, but without endometriosis.
[f] Delivery between 22 + 0–33 + 6 gestational weeks.
[g] Delivery between 34 + 0 to 36 + 6 gestational weeks. Numbers are given as n (%). Some women may have had more than one adverse obstetric outcome. Comparison for each group was made between women with the phenotype to women without. Comparison between groups was made with the chi-square test or Fischer's exact test.
* $P < 0.05$ is considered statistically significant.
