# Supplementary material for: Obstetric outcomes after IVF/ICSI treatment in women with endometriosis and/or adenomyosis diagnosed by ultrasonography: a prospective cohort study
Source: Hum Reprod. 2026 May 27;41(8):1367–76. doi: 10.1093/humrep/deag084 (PMC13429875; doi:10.1093/humrep/deag084)
Supplement: deag084_Supplementary_Table_S3 [file deag084_supplementary_table_s3.pdf]

Human Reproduction, 2026, 1–1
https://doi.org/10.1093/humrep/deag084
Supplementary Data

human reproduction

OXFORD

Supplementary Table S3. Adverse pregnancy outcomes in women with endometriosis and/or adenomyosis, who did or did not undergo treatment with ultralong Gonadotropin releasing hormone treatment.

	Ultralong downregulation, n = 16	No ultralong downregulation, n = 12	*P*-value
Preterm birth	3 (18.8)	1 (8.3)	0.613
Late preterm	2 (12.5)	1 (8.3)	1.0
Caesarean section	3 (18.8)	5 (41.7)	0.231
Placenta previa	2 (12.5)	2 (16.7)	1.0
Small for gestational age	3 (18.8)	2 (16.7)	1.0
Hypertensive disorders of pregnancy	3 (18.8)	4 (33.3)	0.418
Pregnancy induced hypertension	1 (6.3)	3 (25.0)	0.285
Preeclampsia	2 (12.5)	1 (8.3)	1.0
Antepartum hemorrhage	2 (12.5)	5 (41.7)	0.103
Postpartum hemorrhage	0	1 (8.3)	0.429
Diabetes	2 (12.5)	0	0.492
Pelvic pain	2 (12.5)	2 (16.7)	1.0

Numbers are given as n (%). Comparison was made with the chi-square test or the Fischer's exact test. $P < 0.005$ was considered statistically significant.
